# Visualizing artery‐specific blood flow patterns above the circle of Willis with vessel‐encoded arterial spin labeling

**DOI:** 10.1002/mrm.27507

**Published:** 2018-10-25

**Authors:** Thomas W. Okell, Meritxell Garcia, Michael A. Chappell, James V. Byrne, Peter Jezzard

**Affiliations:** ^1^ Wellcome Centre for Integrative Neuroimaging FMRIB Division, Nuffield Department of Clinical Neurosciences University of Oxford Oxford United Kingdom; ^2^ Division of Diagnostic and Interventional Neuroradiology, Department of Radiology, Clinic for Radiology and Nuclear Medicine University of Basel Basel Switzerland; ^3^ Nuffield Department of Surgical Sciences University of Oxford Oxford United Kingdom; ^4^ Institute of Biomedical Engineering, Department of Engineering Sciences University of Oxford Oxford United Kingdom

**Keywords:** arteriovenous malformation, dynamic angiography, vascular territory imaging, vessel‐encoded pseudocontinuous arterial spin labeling (VEPCASL), vessel‐selective

## Abstract

**Purpose:**

To establish the feasibility of using vessel‐encoded pseudocontinuous arterial spin labeling (VEPCASL) for noninvasive vascular territory imaging (VTI) and artery‐specific dynamic angiography of a large number of arterial branches above the circle of Willis within a clinically feasible scan time.

**Methods:**

3D time‐of‐flight angiography was used to select a labeling plane and establish 7 pairs of encoding cycles. These were used for VEPCASL VTI and dynamic 2D angiography (8 min and 3 min acquisition times, respectively) in healthy volunteers, allowing the separation of signals arising from 13 arterial branches (including extracranial arteries) in postprocessing. To demonstrate the clinical potential of this approach, VEPCASL angiography was also applied in 5 patients with brain arteriovenous malformation (AVM).

**Results:**

In healthy volunteers, the artery‐specific filling of the vascular tree and resulting perfusion territories were well depicted. SNRs were approximately 5 times higher than those achievable with single‐artery selective methods. Blood supply to the AVMs was well visualized in all cases, showing the main feeding arteries and venous drainage.

**Conclusions:**

VEPCASL is a highly efficient method for both VTI and dynamic angiography of a large number of arterial branches, providing a comprehensive picture of vascular flow patterns and the effect on downstream tissue perfusion within an acceptable scan time. Automation of labeling plane and vessel‐encoding selection would improve robustness and efficiency, and further refinement could allow quantitative blood flow measurements to be obtained. This technique shows promise for visualizing the blood supply to lesions and collateral flow patterns.

## 1 INTRODUCTION

The ability to distinguish the arterial source of blood supply to a specific brain region or lesion is important in a range of diseases, from the study of collateral flow in steno‐occlusive disease^1^ to the assessment of feeding arteries in arteriovenous malformations/fistulas (AVMs/AVFs)^2^ or brain tumors, such as meningiomas. Conventional x‐ray angiography methods rely on the insertion of a catheter and intra‐arterial contrast agent injection. Whilst yielding high spatial and temporal resolution, the procedure is invasive, time‐consuming, and carries some risks to the patient.^3^

Arterial spin labeling (ASL)^4^, ^5^ is an MRI‐based technique that can be used to obtain vessel‐selective information noninvasively and without contrast agents. Single‐artery selective ASL techniques^6^, ^7^, ^8^ label blood flowing in individually targeted arterial branches of interest.^9^, ^10^, ^11^ Whilst being relatively simple to plan and postprocess, SNR efficiency is reduced compared with conventional nonselective ASL because the labeled blood is restricted to a single artery at a time. Vessel‐encoded pseudocontinuous ASL (VEPCASL) is an alternative approach in which the vessels of interest are encoded by periodically modulating the inversion efficiency across the labeling plane.^12^ A series of images acquired with different spatial modulations can be “decoded” to calculate how much blood arises from each feeding artery in each voxel. If an efficient encoding can be performed, SNR efficiency can equal that of a nonselective ASL acquisition.^12^, ^13^

VEPCASL has previously been used for both angiography^14^ and vascular territory imaging (VTI),^12^ the combination of which allows assessment of both blood flow abnormalities within the vessels and the impact this has on the downstream tissue. However, most work using this technique in the brain has focused on imaging the blood supply arising from the relatively large arteries in the neck. In some situations, more specific information about smaller arterial branches above the level of the circle of Willis may be necessary: for example, in the assessment of the vascular supply to AVMs to aid the planning of embolization therapy.

This presents a challenge due to the large number and more complex geometry of these smaller arteries. In particular, a larger number of vessels requires an increase in the number of vessel‐encoding cycles to uniquely encode each artery of interest. In VEPCASL VTI, this can be achieved by reducing the number of averages of each vessel‐encoding cycle to maintain similar scan times, as has been demonstrated previously.^15^, ^16^ However, for angiographic acquisitions, particularly those that are time‐resolved, each image takes some time to acquire and typically only a single average of each encoding is obtained. The use of a large number of vessel‐encoding cycles would, therefore, lead to unfeasibly long scan times. Investigation of the use of a minimal number of vessel‐encoding cycles for angiographic applications is, therefore, warranted. In addition, recent work has shown that the high SNR efficiency of a balanced steady‐state free precession (bSSFP) readout can be used to accelerate the acquisition of vessel‐selective ASL dynamic angiograms,^17^ which would enable the use of a larger number of encoding cycles within a given scan time.

In this work, we aim to demonstrate the feasibility of using VEPCASL to obtain vessel‐selective dynamic angiograms and vascular territory maps arising from a large number of arterial branches above the circle of Willis using a minimal number of vessel‐encoding cycles in scan times acceptable for compliant patients. In addition, we aim to evaluate the SNR improvement this technique affords relative to that achievable with single‐artery selective methods and demonstrate its clinical potential to visualize the blood supply to brain arteriovenous malformations. This study builds upon work previously presented in abstract form.^18^, ^19^

## 2 METHODS

### 2.1 Healthy volunteer experiments

Five healthy volunteers (1 female; mean age, 30 years) were recruited and scanned using a 3T Verio scanner (Siemens Healthcare, Erlangen, Germany) under a technical development protocol agreed with local ethics and institutional committees. A 32‐channel head coil was used for signal reception and the body coil for RF transmission.

**Figure 1:**
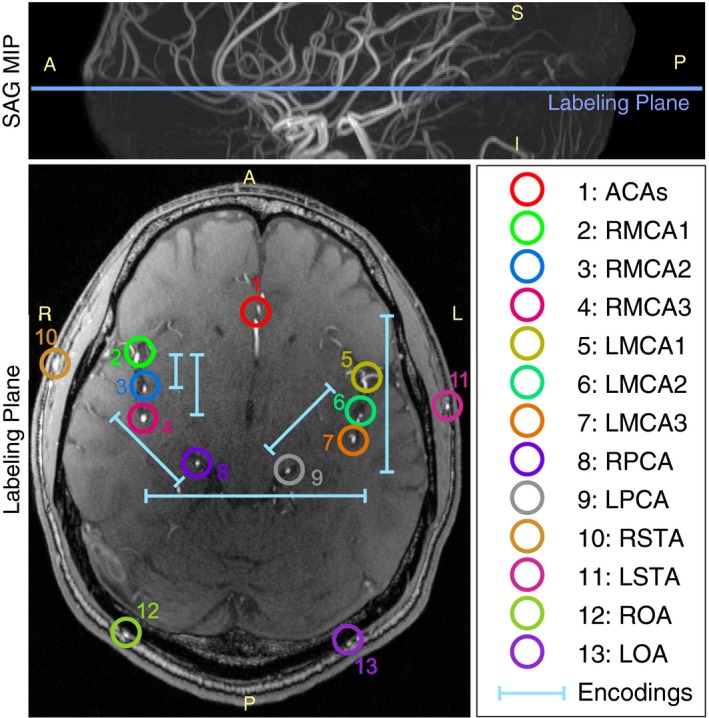
Vessel‐encoding schematic. TOF data from a representative healthy volunteer showing the chosen labeling plane location (top) just above the circle of Willis overlaid on the sagittal oblique maximum intensity projection (MIP). Vessels to be encoded are highlighted within the slice corresponding to the labeling plane (bottom) and numbered as described in the legend. The 6 pairs of vessel‐encodings are also shown, with the ends of each bar representing the tag/control locations of that pair. Note that the encoding function is periodic, so additional tag/control locations will also be present across the slice (not shown for clarity). Orientation labels: A: anterior; P: posterior; S: superior; I: inferior; R: right; L: left

For planning purposes, a 3D time‐of‐flight (TOF) protocol was performed (0.6 × 0.5 × 0.6 mm, 81 slices, acquisition time 3 min) with the bottom edge of the imaging region positioned just below the circle of Willis. The resulting images were then loaded into a 3D viewer to allow selection of a labeling plane at any arbitrary angulation (Figure 1). A plane was selected which contained 3 branches of each middle cerebral artery (MCA), both anterior cerebral arteries (ACAs), and the right and left posterior cerebral arteries (PCAs). The angle between these arterial branches and the labeling plane was maximized where possible.

The artery locations within the labeling plane were recorded and used to establish manually 6 pairs of vessel‐encoding cycles (shown graphically in Figure 1 and listed in Table 1). Nonselective label and control cycles were also applied, giving 14 encoding cycles in total. These encoding cycles were used to perform VEPCASL 2D multislice EPI VTI and cardiac gated dynamic 2D bSSFP angiography. Cardiac gating was achieved using a pulse oximeter. Labeling, background suppression, and readout parameters were similar to previous publications^13^, ^17^ and are listed in Table 2. The imaging regions were positioned parallel to the labeling plane with a separation of more than 15 mm to ensure there was no direct modulation of the static tissue signal from the labeling pulses. Acquisition times were under 8 min and 3 min for VTI and angiography, respectively.

**Table 1:** Vessel‐encoding cycles used for the healthy volunteer study, also shown graphically in Figure 1^a^

Cycle	Encoding direction	Label location	Control location
1	Non‐selective label	–	–
2	Non‐selective control	–	–
3	Left‐right (LR)	RMCA branches	LMCA branches
4	Left‐right (LR)	LMCA branches	RMCA branches
5	Anterior‐posterior (AP1)	ACAs	PCAs
6	Anterior‐posterior (AP1)	PCAs	ACAs
7	Anterior‐posterior (AP2)	Anterior MCA branch	Middle MCA branch
8	Anterior‐posterior (AP2)	Middle MCA branch	Anterior MCA branch
9	Anterior‐posterior (AP3)	Anterior MCA branch	Posterior MCA branch
10	Anterior‐posterior (AP3)	Posterior MCA branch	Anterior MCA branch
11	Diagonal (45, Diag1)	RPCA	Middle RMCA branch
12	Diagonal (45, Diag1)	Middle RPCA branch	RPCA
13	Diagonal (‐45, Diag2)	LPCA	Middle LMCA branch
14	Diagonal (‐45, Diag2)	Middle LMCA branch	LPCA

Abbrevations: ACA, anterior cerebral artery; LMCA, left middle cerebral artery; LPCA, left posterior cerebral artery; RMCA, right middle cerebral artery; RPCA, right posterior cerebral artery.

a Note the paired nature of the encodings, such that the label and control conditions are reversed in the second of each pair.

**Table 2:** Sequence parameters for VEPCASL dynamic angiography and VTI

Parameter	VTI	Dynamic angiography^a^
Tag duration	1400 ms	800 ms
Postlabeling delay	1000 ms	2 ms
Background suppression	Presaturation plus 2 global inversion pulses	Presaturation only
Readout	Gradient echo EPI	bSSFP
In‐plane voxel size	3.4 × 3.4 mm^2^	1.0 × 1.0 mm^2^
Slice thickness	3 mm	50 mm (50 – 100 mm)
Slices	20	1
Flip angle	90	30
Segments^b^	48	14
TE	14 ms	2.0 ms
TR	3350 ms	4.4 ms
Temporal resolution	–	60.9 ms
Time frames	–	12 (28)
Partial Fourier factor	6/8^th^	6/8^th^
Parallel imaging (GRAPPA) factor	1	2
Volumes acquired	140	14 (10‐14)
Acquisition time	7 min 50 s	~2 min 50 s (~4 min)^c^

Abbrevations: VEPCASL, vessel‐encoded pseudocontinuous arterial spin labeling; VTI, vascular territory imaging.

a Where parameters differ for the AVM patient protocol, these are given in parentheses.

b Segments refers to lines of k‐space acquired for each slice and time frame after each ASL preparation.

c Depending on the cardiac cycle and number of vessel‐encodings performed.

### 2.2 AVM patients

To demonstrate the feasibility of this approach in a clinical cohort, 5 patients with brain AVMs (3 male; mean age, 51 years) were scanned as part of their pretreatment assessment. Patient informed consent and institutional ethical and committee approval were obtained in advance for both MRI scans and the use of data from x‐ray digital subtraction angiograms (DSA). As per the healthy volunteer study, TOF angiography was performed for labeling plane selection and vessel localization. The labeling plane was chosen to be close enough to the AVM to identify potential feeding arteries, but sufficiently far to prevent the labeling artifact mentioned above. Due to the variability in AVM locations and feeding vasculature, vessel‐encodings were manually prescribed on a per subject basis (an example is given in Supporting Information Figure S1, which is available online). These varied between 5 and 7 pairs of cycles, depending on the number of arterial branches present within the labeling plane, including nonselective label and control cycles.

VEPCASL 2D dynamic angiograms were obtained in oblique transverse, coronal, and sagittal acquisitions, oriented orthogonally to the labeling plane. Sequence parameters were very similar to the healthy volunteer protocol (see Table 2), except the slab thickness was varied in the range 50‐100 mm to cover the AVM and the acquisition window was extended to ~1700 ms (depending on the cardiac cycle) to allow visualization and assessment of venous drainage, leading to an acquisition time of approximately 4 min per scan. Because the signals of interest were expected to remain intravascular in these patients, no equivalent VTI was performed. X‐ray DSA data from studies performed by injections of radiographic contrast media in the carotid and vertebral arteries, as part of the clinical workup of these patients, were also available for comparison. However, due to the differing image orientations and limited number of patients, only qualitative comparisons could be made between the 2 modalities.

### 2.3 Image analysis

Separation of vascular components in the VEPCASL data was performed using a maximum a posteriori solution^20^ to the Bayesian framework for vessel‐encoded ASL analysis^16^ with 1 vessel per class. This procedure is initialized with the vessel locations from the TOF images, but can account for rigid in‐plane subject motion between the planning and VEPCASL scans. It also improves the SNR of the resulting images compared with a conventional matrix pseudo‐inversion approach,^12^ while still allowing probabilistically for mixed blood supply. Upon inspection of the healthy volunteer data, it was apparent that branches of the extracranial arteries also contributed signal to the imaging region, particularly in the angiographic acquisition, so these were also identified within the TOF images and included in the analysis: specifically, the right and left superficial temporal arteries (STAs) and occipital arteries (OAs). This gave a total of 13 arteries plus static tissue to separate. The number of arterial components varied between 5 and 10 across the AVM patients. In these data sets, the extracranial arteries were only included in the analysis if they appeared to contribute any significant signal to the imaging region. It should be noted that no blood flow quantification was performed in this study, although it is expected this should be possible in future work (see the Discussion section).

### 2.4 SNR comparison

Using the healthy volunteer data, the SNR benefit of VEPCASL relative to single‐artery selective approaches can be evaluated. This is because if single‐artery selective data were acquired with matched scan time and imaging parameters, this would allow the targeting of 7 arterial branches with dedicated tag and control cycles, or 13 if a single control image were used, with the same number of averages as the VEPCASL protocols. Each vessel‐specific image would then be derived from 2 cycles of data. Given that the inversion efficiency obtained in single‐artery selective techniques is equal to or lower than that achievable with conventional PCASL,^7^, ^8^ the SNR obtained from subtraction of the nonselective control and label cycles acquired here represents an upper bound for the SNR of a single‐artery selective image. Therefore, the ratio of the SNR obtained from just the nonselective data to that of the VEPCASL vessel‐specific images demonstrates the minimum SNR improvement over single‐artery selective methods that VEPCASL can achieve.

To evaluate SNR, data from the nonselective cycles were subtracted to give a conventional ASL image. This was used to generate a mask by thresholding at a factor of 0.3 times the 99th percentile intensity, which was found empirically to include the majority of the vessels (for angiography) and gray matter (for VTI) while excluding the background. The SNR of the nonselective data was then calculated as the mean signal within this mask divided by the SD within a background region. For the vessel‐encoded data, SNR was calculated for each vessel‐selective map separately and the same masks were used for the calculation. However, because each vessel only supplies part of the brain, the mask was split such that the mean signal was calculated using only voxels in which that artery was the dominant component (i.e., contributed the largest signal), as per previous work.^13^ The mean SNR across all vessel‐specific maps was then calculated, weighted by the number of voxels within each mask. Statistical significance between the SNR of the vessel‐encoded and nonselective data was established using a paired t‐test across subjects for the VTI and angiographic data separately.

## 3 RESULTS

**Figure 2:**
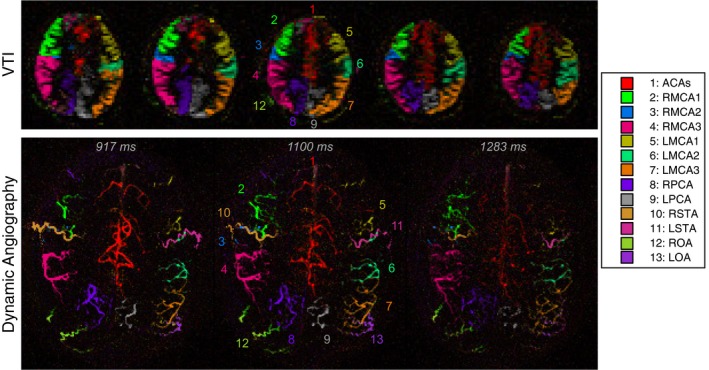
Example VEPCASL data from the same healthy volunteer as Figure 1. Selected slices of VTI data (top) and selected frames of dynamic angiography (bottom) are shown, with color and numbering used to show the arterial origin of the blood signal, as shown in the legend. Clear delineation of the vascular territories arising from these arterial branches is seen. Signal from the extracranial arteries is particularly apparent in the angiographic images. Times shown above the angiographic frames are relative to the start of the VEPCASL labeling. All frames of the VEPCASL angiogram can be viewed in Supporting Information Video S1

Example VEPCASL vascular territory maps and angiographic images from 1 healthy volunteer are shown in Figure 2 and Supporting Information Video S1. Clear separation of the signals arising from the different arterial branches was seen, matching the expected distribution,^21^ and there was good correspondence between the VTI and angiographic images. Extraparenchymal signal from the extracranial arteries was particularly apparent in the angiographic data and was well separated from the other arterial branches despite not being explicitly included in the planning of the encodings. The dynamic flow of blood through the arterial tree was also well visualized in the angiographic images.

**Figure 3:**
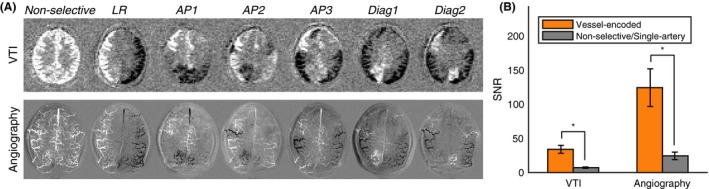
SNR comparison. (A) Subtractions of pairs of encodings for both VTI (top) and angiography (bottom) demonstrate that all arteries contribute significant signal across most of the applied vessel‐encodings, giving a considerable SNR boost when they are combined to generate vessel‐specific maps. (B) This is apparent in the mean SNR results across all subjects, where the vessel‐encoded data have significantly higher SNR (*P < 0.001) than just the nonselective tag/control data, which is an upper bound for the SNR that could be achieved by single‐artery selective methods

Figure 3 shows examples of direct subtraction within each pair of encodings, highlighting the significant signal contribution made by most arteries to each encoding. This results in a considerable SNR improvement when the images are combined into vessel‐specific maps. The mean SNR of the nonselective data, which is an upper bound on that achievable with single‐artery selective methods, was significantly lower (*P* < 0.001) than that of the vessel‐encoded data. The mean SNR ratio of vessel‐encoded to nonselective acquisitions was 4.8 ± 0.9 for VTI and 5.2 ± 1.4 for angiography.

**Figure 4:**
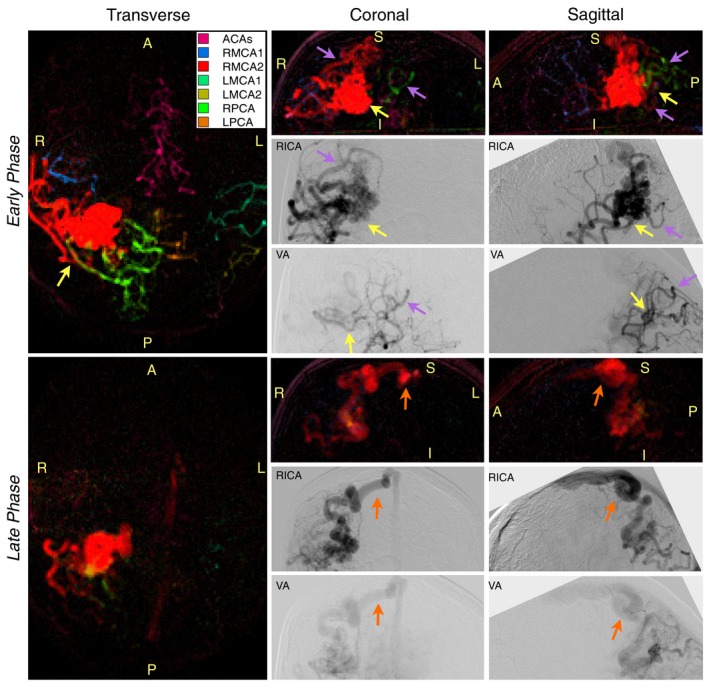
Selected frames from VEPCASL angiograms in oblique transverse, coronal, and sagittal orientations from 1 AVM patient, showing both early and late phases (917 ms and 1466 ms after the start of labeling, respectively). X‐ray DSA images following injection into the right internal carotid artery (RICA, which supplies the RMCA) and 1 vertebral artery (VA, which supplies the PCAs) are also shown for comparison. Despite differences in image orientations and timings, there is good qualitative correspondence between the 2 modalities (purple arrows). In both cases, it is clear the AVM is mainly supplied by the RPCA and branches of the RMCA (yellow arrows). Comparable visualization of venous drainage is also observed (orange arrows). Note that the color scheme used here differs from Figure 2 due to the different number of arterial components (see legend), and the sagittal x‐ray images have been rotated to try to match the VEPCASL image orientation more closely. All frames of the VEPCASL data can be viewed in Supporting Information Videos S2, S3, and S4

VEPCASL angiograms of 1 AVM patient are shown in Figure 4 and Supporting Information Videos S2, S3, and S4. The abnormal vessels are clearly depicted, and there is a good separation of the vascular components, demonstrating the primary feeding arteries to the AVM. In later frames, venous drainage, normally absent from ASL angiograms, is also well visualized. Despite differences in image orientation and bolus timing, there is good qualitative agreement between VEPCASL angiography and x‐ray DSA in terms of the vessel morphology, feeding‐artery identification, and venous drainage. VEPCASL angiograms in the other 4 AVM patients were of similar quality, as shown in Supporting Information Figure S2.

## 4 DISCUSSION

In this study, we have demonstrated the feasibility of using VEPCASL to obtain vascular territory maps and vessel‐selective dynamic angiograms arising from up to 13 arterial branches above the circle of Willis in healthy volunteers and AVM patients within a clinically feasible scan time. The use of vessel‐encoding means that most arteries contribute significant amounts of signal to each encoding cycle, boosting the SNR by a factor of 5 compared with targeting 1 vessel at a time and thereby keeping the scan time low, even for a large number of arterial branches. The use of bSSFP for the angiographic readout gives a large increase in both imaging speed and SNR compared with spoiled gradient echo techniques,^17^ allowing the encoding of large numbers of vessels while keeping acquisition time below 4 min. The longer VTI scan time (8 min) used here ensured high SNR within the relatively thin slices acquired (3 mm), but acquisition time could be significantly reduced while maintaining SNR by increasing the slice thickness.

The Bayesian analysis technique used in this study can account for subject motion between the planning and acquisition and allows the separation of signals from different arteries even if the encoding matrix is rank deficient by considering subsets of this matrix.^16^ This allowed for the separation of signals from the extracranial arteries in the analysis even though they were not explicitly included in the planning process, and also improved SNR. It is interesting to note that using a matrix inversion approach with an ideal encoding scheme the SNR efficiency of a VEPCASL acquisition should be equal to that of a conventional nonselective acquisition.^12^ Therefore, in this study, in the case of ideal vessel‐encodings, we would expect that the VEPCASL data (constructed from 14 encoding cycles) would have higher SNR than the nonselective images (constructed from 2 cycles) by a factor of √(14/2) = 2.6. However, the measured SNR increase was approximately a factor of 5. This is a consequence of the Bayesian analysis approach that limits the number of arteries expected to contribute signal to each voxel. This is much like a sparsity constraint used in compressed sensing,^22^ where here we expect the vector of signal contributions from all arteries in a given voxel to be sparse (i.e., most entries equal to 0). In both cases, this has a denoising effect, boosting the SNR above that expected for a simple matrix inversion analysis, in which it is implicitly assumed that all arteries can contribute to each voxel.

The VEPCASL angiograms acquired in AVM patients demonstrate that this technique has clinical potential. The derived information could prove useful in the morphological evaluation of AVMs pretherapeutically, which could aid the planning of embolization therapy, and also in postembolization assessment, reducing the need for invasive x‐ray DSA procedures before an intervention and in follow‐up evaluation. Only minimal motion artifacts were observed across all 5 clinical subjects, showing that the scan time required for VEPCASL angiography is well tolerated in this group of patients. The qualitative similarity to x‐ray DSA data is also encouraging, although differences in image orientation, the lack of x‐ray DSA data with contrast injections into the same arterial branches, and the small number of patients studied here precludes a rigorous comparison of the 2 techniques.

**Figure 5:**
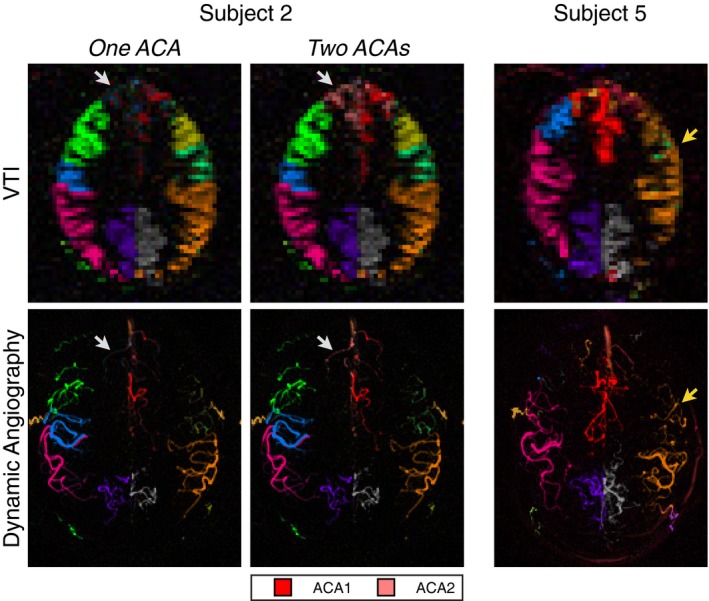
Challenges with labeling above the circle of Willis. VTI (top) and angiography (bottom) images from subjects 2 and 5 of the healthy volunteer study, which show good general separation of vascular territories. However, in subject 2 (left) the ACAs were originally treated as a single vessel component, which led to poor decoding of some ACA branches (white arrows). The separation of the initially combined ACAs into 2 branches in the analysis resulted in improved vascular territory maps. In subject 5 (right), several of the MCA branches identified at the labeling plane did not appear in the final images (yellow arrows). On closer inspection of the TOF angiogram it became apparent that these branches turn inferiorly after passing through the labeling plane and so do not contribute any significant blood supply to the imaging region. Color‐coding is identical to Figure 2, except the new ACA branch in subject 2, which is shown in the legend

The main difficulty encountered in this study was the requirement to manually identify a single labeling plane in which to encode the tortuous vessels above the circle of Willis. This led to the suboptimal identification of relevant arterial branches in some healthy volunteers, as shown in Figure 5. In the first example, the bilateral ACAs were originally treated as a single artery, but this led to poor decoding of some of the blood flow within their distribution territories. Retrospective examination of the labeling plane and the addition of a second ACA into the analysis resulted in more accurate vascular territory maps. In the second example, most of the blood within each MCA territory arose from a single MCA branch identified at the labeling plane. Upon inspection of the TOF data, it became apparent that some of the identified MCA branches flowed superiorly before turning inferiorly, and, therefore, did not contribute significantly to blood flow within the imaging region. It is, therefore, clear that care needs to be taken when considering which arterial branches to encode. In addition, the vessel‐encodings were prescribed manually in this study, which was time‐consuming and may have resulted in suboptimal encodings, particularly for the extracranial arteries, which were not included in the planning process, and for the MCA branches, if they were not symmetric about the midline within the labeling plane.

Another VEPCASL approach involves using a large number of randomly chosen encoding cycles and the subsequent identification of the feeding arteries in postprocessing.^15^ This simplifies the scan setup, but results in a theoretical SNR penalty of √2 compared with an ideal encoding scheme,^15^ which may be more severe for vessels that are not well separated.^23^ In addition, the use of a large number of encoding cycles is not possible for angiography where each image is time‐consuming to produce.

An alternative approach for these kinds of acquisitions is single‐artery selective ASL,^6^, ^7^, ^8^ which has also been used successfully above the circle of Willis in healthy volunteers and AVM patients.^9^, ^10^, ^11^ These techniques have the advantage of being relatively simple to prescribe and postprocess, and also allow the labeling plane to be reoriented to best suit each targeted artery. Some restriction on the angulation of these labeling planes remains, because they must not coincide with the imaging region, but this removes the requirement that a single plane has to be identified in which all arteries run approximately in the through‐plane direction. However, there are some disadvantages to this approach. Reducing the size of the labeling spot improves vessel‐selectivity but also reduces labeling efficiency.^7^, ^8^ Therefore, a compromise must be reached which introduces the potential for partial labeling of nearby arteries^24^ and consequently inaccurate vessel‐selective images. To perform the same experiment shown here with single‐artery selective methods 26 images would need to be acquired (if separate control images were obtained for each artery), almost doubling the total scan time.

Alternatively, a single control image could be used, so only 14 images would be required as in this work, but this increases the likelihood of partial labeling of nearby arteries.^25^ In addition, the SNR of the resulting vessel‐specific maps would be similar to or lower than the nonselective data shown here, giving a considerable loss of SNR compared with the vessel‐encoded approach: approximately a factor of 5. This could be mitigated to some degree in cases where only a few arterial branches are of interest, because the SNR efficiency loss of single‐artery methods scales with the square root of the number of targeted vessels.^26^ However, in the AVM data shown here and in another study,^10^ several arterial branches often supply the lesion, and nearby branches may also need to be targeted to exclude their involvement.

Similar numbers of vessels would likely be of interest in other clinical scenarios, such as the assessment of tumors or collateral flow, if studied at this level in the brain. Therefore, vessel‐encoding is particularly appealing if information about a large number of arteries is desired and a suitable labeling plane can be identified. In cases where only a small number of arterial branches are of interest or SNR efficiency and scan time are not primary concerns, single‐artery methods may be simpler and more flexible to perform.

In future work, several improvements could be made to the vessel‐encoding process. In this study, paired encoding cycles were used, as in several previous studies.^13^, ^14^, ^15^ Although not strictly necessary to decode the signals from each artery, the subtraction of pairs of cycles allows direct examination of the impact of each encoding (see Figure 3). However, the use of unpaired encodings could provide more unique information within a given number of encoding cycles, thereby improving the rank and condition number of the encoding matrix. In addition, optimization of the encoding scheme could be performed using an automated algorithm,^23^ improving SNR efficiency and reducing the required planning time. Extracranial arteries could also be explicitly included in the planning process, which would be particularly useful for angiography where their contribution is more significant, to make sure all vessels are uniquely encoded. An algorithm that could automatically choose an optimal labeling plane location from TOF data would be beneficial, particularly when the vascular anatomy is very tortuous. Finally, the spatial extent of the artifact associated with the labeling plane could be minimized by using stronger PCASL labeling gradients, which could allow labeling closer to a lesion such as an AVM.

Although motion artifacts were not problematic in the AVM patients scanned for this study, for some clinical applications, particularly in acute settings, reduced scan time and sensitivity to motion would be important: the analysis technique used here can correct for in‐plane motion between planning and imaging, but the addition of navigators for prospective motion correction would also be beneficial to minimize the effect of through‐plane motion and intrascan motion artifacts.^27^, ^28^ Acquisition acceleration and extension to a time‐resolved 3D angiographic readout should be possible through more extensive use of parallel imaging,^29^ compressed sensing^22^ and undersampled radial trajectories.^30^, ^31^ Full validation of these techniques against established methods, such as invasive x‐ray techniques, would also be required before widespread clinical adoption. If quantitative information about blood flow is required, a multi‐PLD or longer single‐PLD acquisition would be necessary for perfusion imaging, along with a careful assessment of the PCASL inversion efficiency within the tortuous vessels at this level in the brain. However, we hope that these approaches could ultimately further improve the applicability of VEPCASL imaging for both angiographic and perfusion assessment in patients with vascular lesions, such as AVMs/AVFs, as well as in patients with steno‐occlusive disease or brain tumors.

## Supporting information


**Figure S1** Example of VEPCASL planning for the AVM patient shown in Figure 4. In this case the labeling plane was positioned at a double oblique angle, shown overlaid on the TOF MIPs (left). This plane incorporated the ACAs, two branches of each MCA and the PCAs, which were encoded using five pairs of VEPCASL cycles (right).
**Figure S2** The first frame of the transverse VEPCASL angiograms of the other four AVM patients included in this study. In all cases good separation of arterial components was achieved, showing the main feeding arteries to the AVM. Each patient has a different color coding according to the number of arterial branches of interest in each case, as shown in the legends.

**Video S1** Movie of the VEPCASL dynamic angiography data from a healthy volunteer shown in Figure 2.

**Video S2** Transverse movie of the VEPCASL dynamic angiography data from an AVM patient shown in Figure 4.

**Video S3** Coronal movie of the VEPCASL dynamic angiography data from an AVM patient shown in Figure 4.

**Video S4** Sagittal movie of the VEPCASL dynamic angiography data from an AVM patient shown in Figure 4.
