# Supplementary material for: Visualizing artery‐specific blood flow patterns above the circle of Willis with vessel‐encoded arterial spin labeling
Source: Magn Reson Med. 2018 Oct 25;81(3):1595–604. doi: 10.1002/mrm.27507 (PMC6492185; doi:10.1002/mrm.27507)
Supplement: Supplementary file 1 — Figure S1 Example of VEPCASL planning for the AVM patient shown in Figure 4. In this case the labeling plane was positioned at a double oblique angle, shown overlaid on the TOF MIPs (left). This plane incorporated the ACAs, two branches of each MCA and the PCAs, which were encoded using five pairs of VEPCASL cycles (right). Figure S2 The first frame of the transverse VEPCASL angiograms of the other four AVM patients included in this study. In all cases good separation of arterial components was achieved, showing the main feeding arteries to the AVM. Each patient has a different color coding according to the number of arterial branches of interest in each case, as shown in the legends. [file MRM-81-1595-s001.pdf]

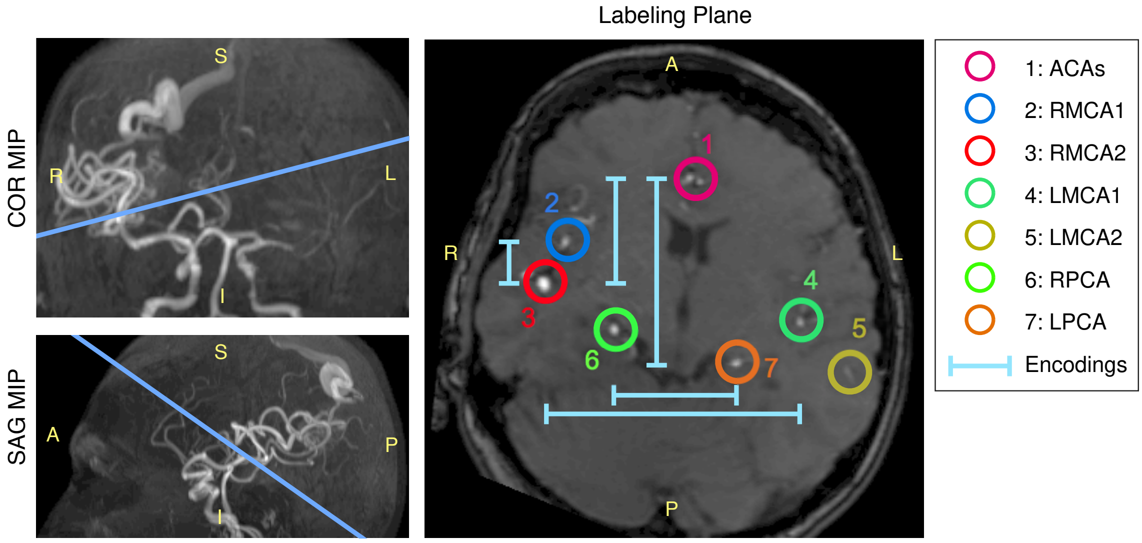

Supporting Information Figure S1: Example of VEPCASL planning for the AVM patient shown in Figure 4. In this case the labeling plane was positioned at a double oblique angle, shown overlaid on the TOF MIPs (left). This plane incorporated the ACAs, two branches of each MCA and the PCAs, which were encoded using five pairs of VEPCASL cycles (right).

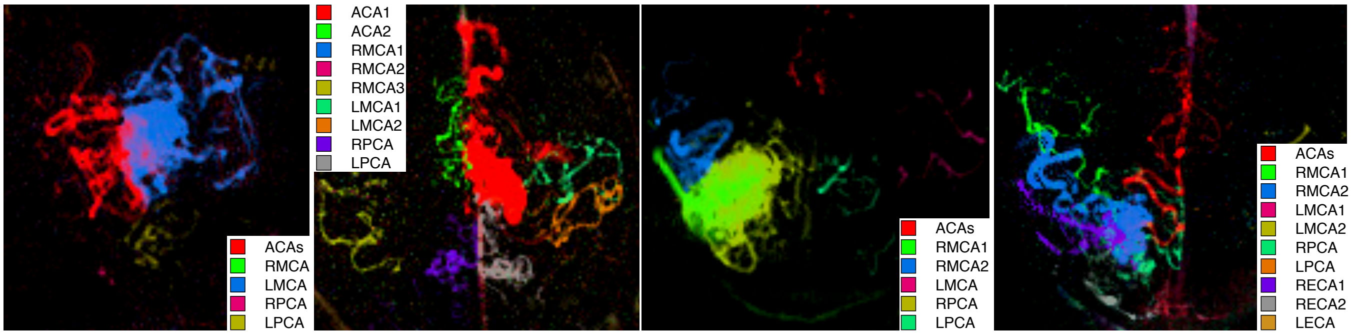

Supporting Information Figure S2: The first frame of the transverse VEPCASL angiograms of the other four AVM patients included in this study. In all cases good separation of arterial components was achieved, showing the main feeding arteries to the AVM. Each patient has a different color coding according to the number of arterial branches of interest in each case, as shown in the legends.
